# Technical Considerations in Rehabilitation of an Edentulous Total Glossectomy Patient

**DOI:** 10.1155/2012/125036

**Published:** 2012-02-28

**Authors:** Pravin Bhirangi, Priyanka Somani, K. P. Dholam, Gurmeet Bachher

**Affiliations:** Department of Dental and Prosthetic Surgery, Tata Memorial Hospital, Mumbai 400 012, India

## Abstract

The technician by virtue of his profession plays an important role in fabricating silicone tongue prosthesis for a total glossectomy patient. The technician, with his skills and specialized knowledge in handling material, plays a valuable role as a member of the oncology team. A patient with total glossectomy can be rehabilitated by silicone tongue prosthesis as an aid to improve his speech and swallowing. This paper describes the technical steps involved in fabricating a silicone tongue prosthesis for an edentulous total glossectomy patient.

## 1. Introduction

Oncologic management of advanced carcinoma of the tongue is a difficult medical problem that can create serious treatment dilemmas. Often criticized as a treatment option because of the severe functional sequelae, total glossectomy may sometimes be the treatment of choice.

Tongue prosthesis occupies the space in the floor of the oral cavity. It provides the patient with a platform for directing food into the esophagus and aids in speaking.

This paper describes the technical steps involved in prosthetic management of an edentulous patient (EP) who underwent total glossectomy with a prosthesis made of silicone tongue (fabricated with molloplast-B) attached to mandibular complete denture.

EP reported with ulceroproliferative growth on tongue (T_4_N_2a_M*_x_*). He underwent near total glossectomy with modified neck dissection with pectoralis major myocutaneous flap reconstruction. Postoperatively, he received radiotherapy (4600 Gy) only.

**Figure 1:**
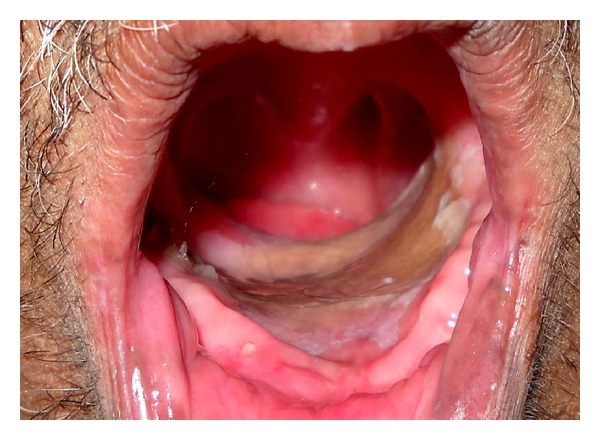
Preoperative intraoral view.

**Figure 2:**
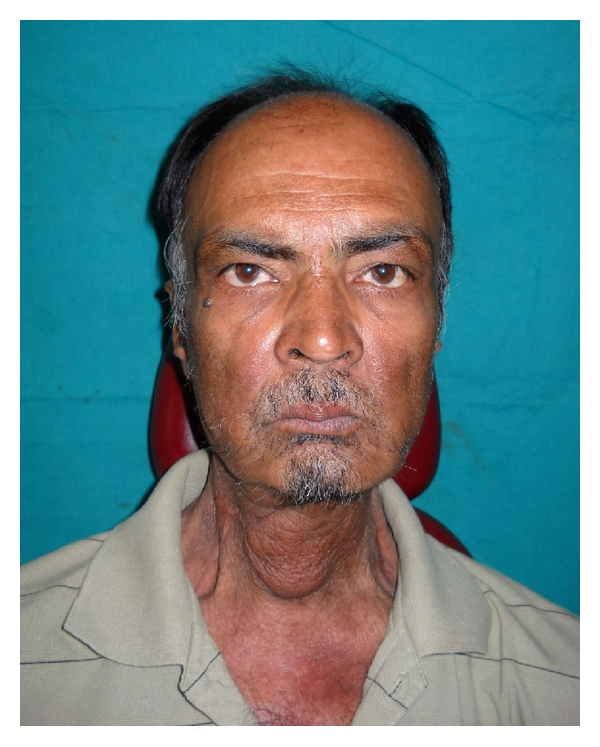
Preoperative extraoral view.

The patient complained of difficulty in eating and swallowing with no complaint of aspiration. He was on liquid diet without ryles tube. After completion of one year of radiation, the intraoral tissues healed to withstand prosthetic intervention on clinical examination. In view of total surgical excision of the tongue and completely edentulous state of the upper and lower jaws, tongue prosthesis was planned for the patient (Figures 1 and 2).

## 2. Procedure

**Step 1**

Mucocompressive impression was made of an upper edentulous arch using impression compound and mucostatic impression was made of lower edentulous arch and floor of the mouth using irreversible hydrocolloid impression material by a maxillofacial prosthodontist. The disinfected impressions were then sent to the dental laboratory.

**Step 2**

The diagnostic casts/primary casts were obtained. Custom trays were fabricated on upper and lower edentulous primary casts with autopolymerizing acrylic resin.

**Step 3**

**Figure 3:**
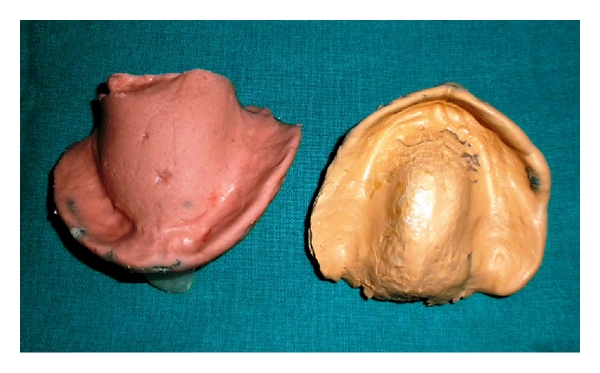
Final Impression.

Border molding using low fusing impression compound was done, and secondary impressions of upper and lower edentulous arch with zinc oxide eugenol impression paste using selective pressure impression technique were made (Figure 3). Stock tray was then used to make a pick-up impression of this (only mandibular arch) and to record floor of the mouth together as a single impression (irreversible hydrocolloid impression).

**Step 4**

The impressions were disinfected and poured with type III dental stone to obtain master cast.

**Step 5**

On the master cast, temporary record bases were made using autopolymerizing acrylic resin, which were tried in patient's mouth to check retention and stability.

**Step 6**

On the temporary record bases, occlusal rims were made to record maxilla-mandibular relation and the teeth arrangement was done following recording of jaw relation.

**Step 7**

**Figure 4:**
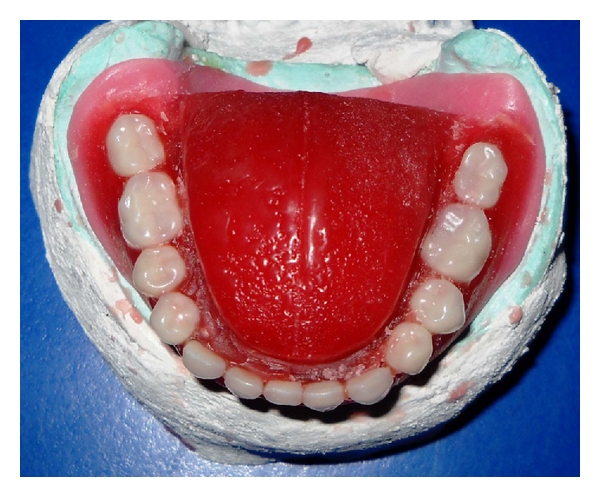
Wax up trial of tongue prosthesis.

Wax up of tongue prosthesis [1] was done on the temporary record bases, which was contoured in the shape of a tongue that conforms to oral cavity dimensions with rounded edges (Figure 4).

The tongue tip was arched inferiorly to approximately a 15-degree angle, and the entire pattern was then arched slightly to form the highest point at the anterior one third.

Wax pattern was then folded to form a wide central V-shaped angle (approximately 160 degrees). The wax was reduced 4 to 5 mm of thickness at the base and the posterior two thirds.

The wax pattern was finally sealed to the lower trial denture.

**Step 8**

**Figure 5:**
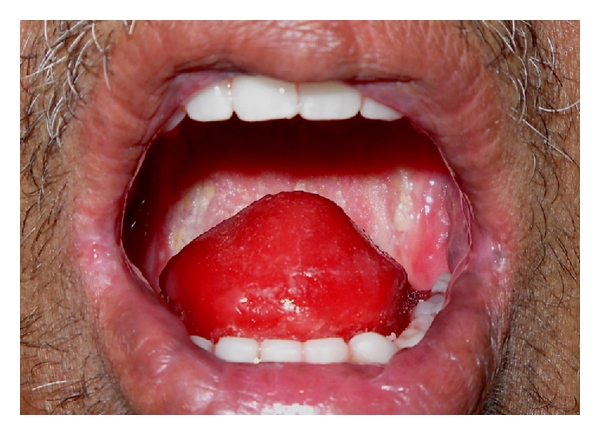
Wax up trial of tongue prosthesis in the patient's mouth.

The waxed up prosthesis was then tried in patients mouth. (Figure 5) The speech therapist evaluated the speech articulation with the waxed up tongue (WT) for maximum contact of the dorsum with palate for optimum articulation.

**Step 9**

**Figure 6:**
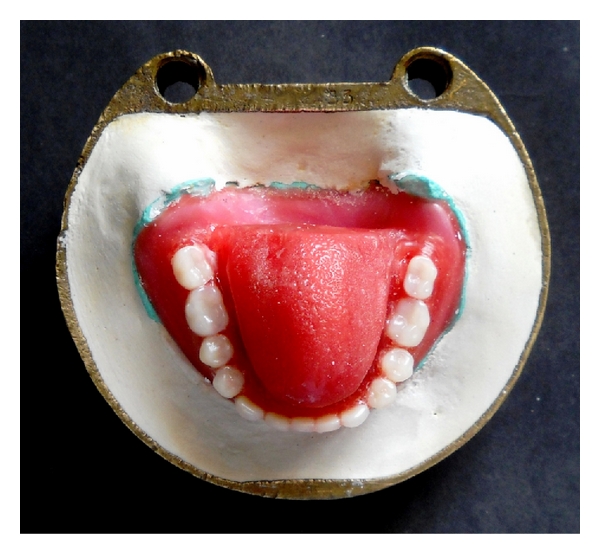
Flasking of the tongue prosthesis.

**Figure 7:**
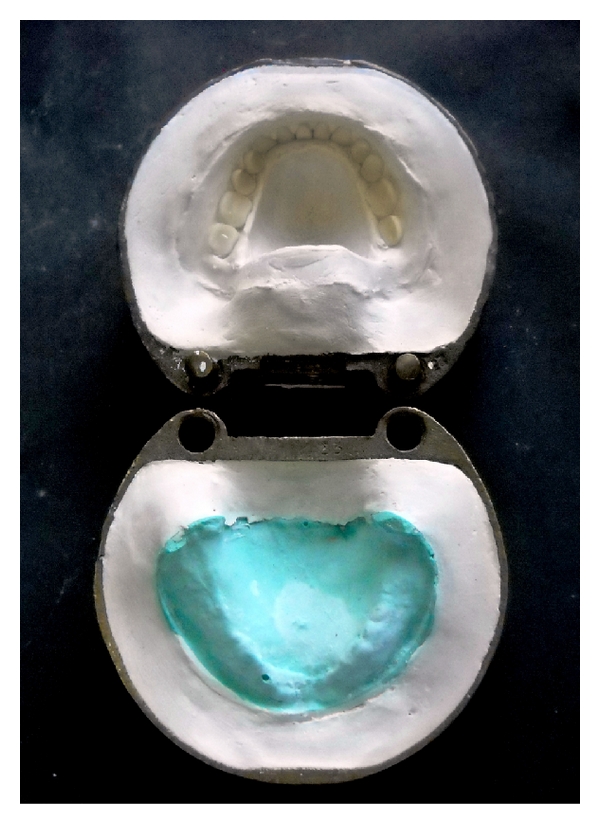
Dewaxing of the tongue prosthesis.

Processing of silicone tongue prosthesis: flasking and dewaxing was done for the waxed up upper and lower prosthesis in conventional manner (Figures 6 and 7).

**Figure 8:**
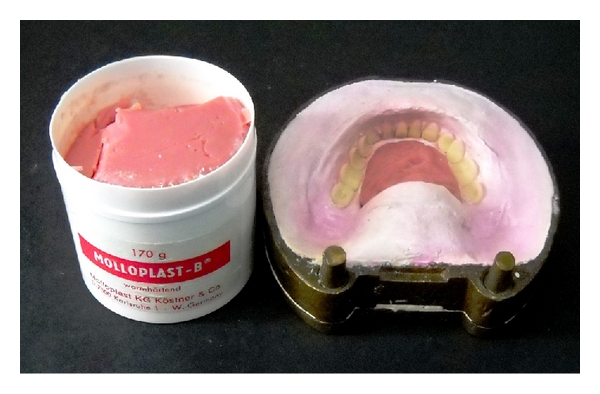
Packing of Molloplast-B material.

At the packing stage, the heat temperature vulcanizing silicone (Molloplast-B, Regneri GmbH & Co.KG, W-Germany) was packed in tongue space (Figure 8).

Trial closure was done and excess molloplast material was removed.

**Figure 9:**
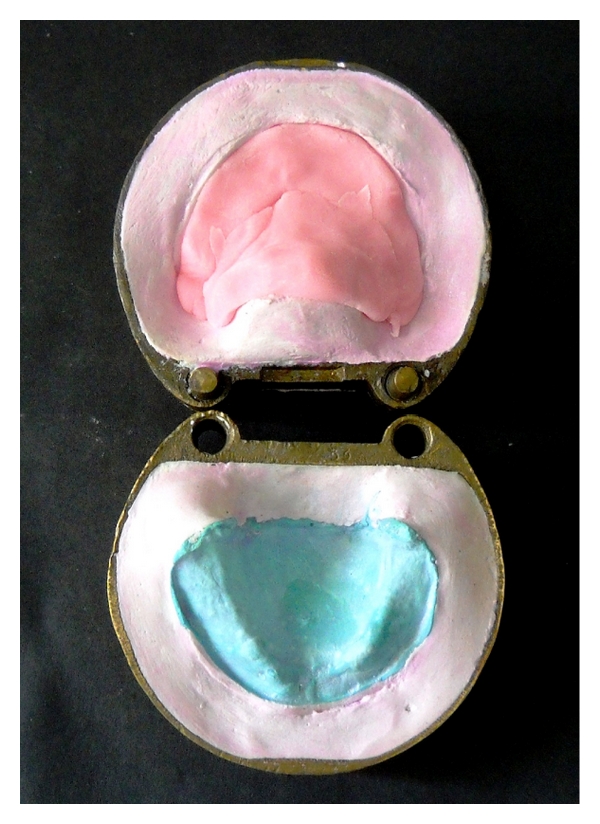
Packing of heat cure acrylic resin.

Heat cure denture base resin was packed in edentulous ridge space (Figure 9).

The flask was closed and kept for overnight bench curing.

Curing of the prosthesis was done as per manufacturer instructions.

**Figure 10:**
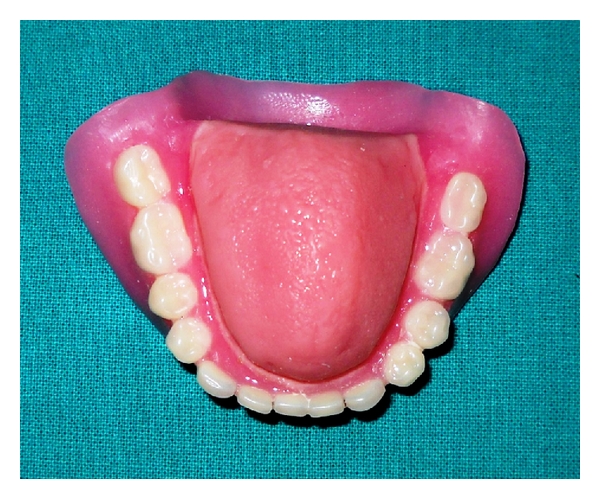
Final tongue prosthesis.

**Figure 11:**
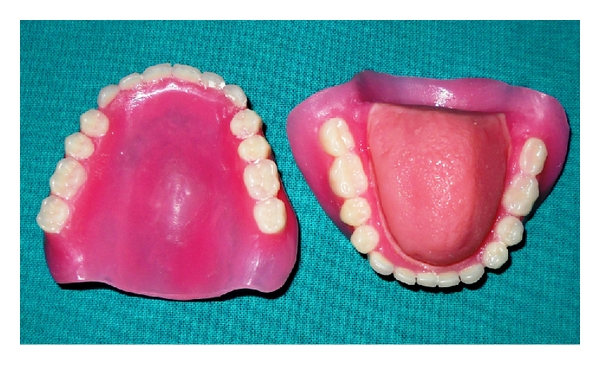
Final tongue prosthesis.

After deflasking, the prosthesis was trimmed, finished, and polished (Figures 10 and 11).

## 3. Discussion

Swallowing and speaking by prosthodontic management of glossectomy patient is a difficult undertaking for both the prosthodontist and the patient. Particular considerations should be given to the patient's chief complaints when planning treatment for the glossectomy patient. A patient may be able to accommodate to some dysfunction without prosthetic support, while desiring prosthetic treatment helps to improve or correct other specific problems [2].

A wide buccolingual table, an occlusal table height matched to that of the tongue body, and a closely adhering tongue and lingual flange are effective means of preventing the food from dropping to the oral floor, keeping the food on the occlusal table, and crushing the food.

When constructed in a systematic manner with the assistance of a speech pathologist, the mandibular tongue prosthesis can achieve the following [3]:

1. reduction in the size of the oral cavity, thereby improving resonance characteristics,
2. direction of food into the esophagus with the aid of a trough carved into the prosthetic tongue,
3. protection of the underlying fragile tissue,
4. development of a surface for the residual tongue tissue to contact during speech and swallowing,
5. improvement in appearance and psychosocial adjustment.

**Figure 12:**
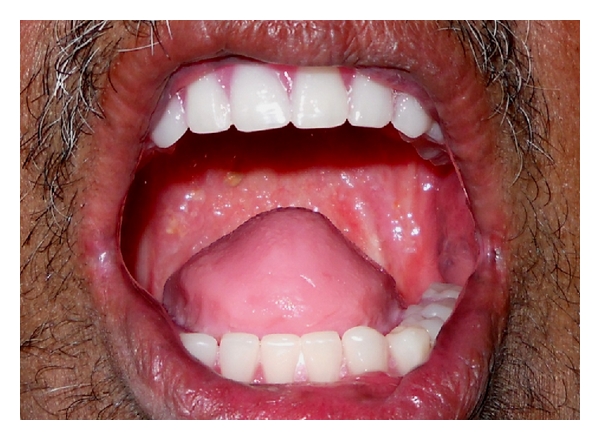
Tongue prosthesis in the patient's mouth.

**Figure 13:**
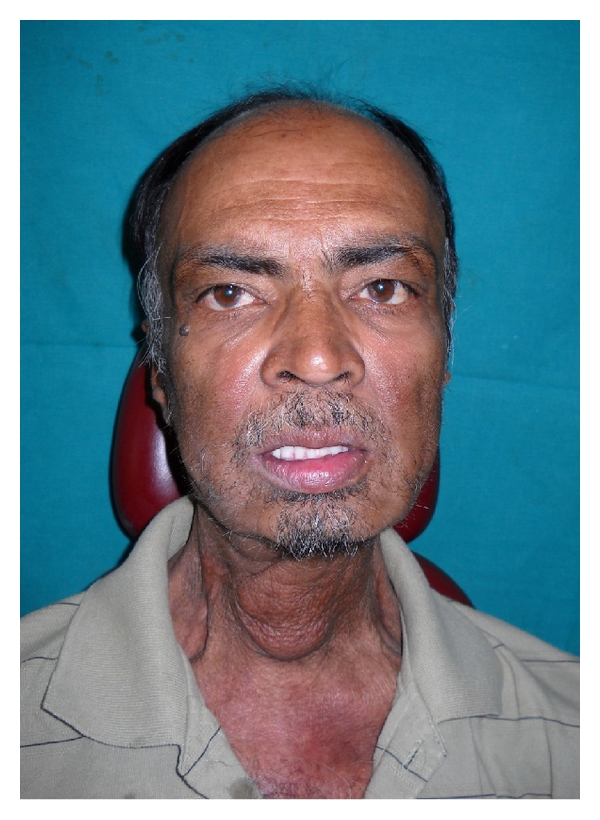
Postoperative extraoral view.

In this EP, after fabrication of the tongue prosthesis swallowing improved. Liquid diet was replaced with semisolids without difficulty and apparent aspiration (Figures 12 and 13).

Improvement in speech was also observed. Without tongue prosthesis, he was substituting labiodentals and vowels for fricative and palatal sounds. After using prosthesis for six months, fricative and palatal sounds improved audibly.

The material used to fabricate tongue prosthesis, silicone (Molloplast-B, Molloplast, Regneri GmbH @ Co, W- Germany), permanent soft relining material, has several advantages [4]:

- single component, ready to use eliminating mixing and dosing errors,
- easy processing,
- can be polymerized simultaneously with acrylic,
- stands the influences of oral environment without deterioration,
- nonirritant and tissue compatible,
- odourless and tasteless.

## 4. Summary

The paper describes technical steps for the prosthetic rehabilitation of an edentulous glossectomy patient, in which treatment dentures were constructed with mandibular tongue prosthesis and the appropriate denture form and occlusion was established.

The prosthetic tongue may not replace the intricately mobile structure of the tongue, which is capable of infinite movements in swallowing and speech. The silicone tongue prosthesis does provide glossectomy patient with a certain degree of comfort and function.
